# Selective episiotomy vs. implementation of a non episiotomy protocol: a randomized clinical trial

**DOI:** 10.1186/1742-4755-11-66

**Published:** 2014-08-14

**Authors:** Inês Melo, Leila Katz, Isabela Coutinho, Melania Maria Amorim

**Affiliations:** 1 Instituto de Medicina Integral Prof. Fernando Figueira, Recife, PE, Brazil; 2 Department of Obstetrics and Gynecology, Federal University of Campina Grande, Campina Grande, PB, Brazil

**Keywords:** Episiotomy, Vaginal delivery, Perineum, Randomized controlled trial

## Abstract

**Background:**

World Health Organization (WHO) recommends that the episiotomy rate should be around 10%, which is already a reality in many European countries. Currently the use of episiotomy should be restricted and physicians are encouraged to use their clinical judgment to decide when the procedure is necessary. There is no clinical evidence corroborating any indication of episiotomy, so until the present moment it is not yet known whether episiotomy is indeed necessary in any context of obstetric practice.

**Objectives:**

To compare maternal and perinatal outcomes in women undergoing a protocol of not performing episiotomy versus selective episiotomy.

**Methods/Design:**

An open label randomized clinical trial will be conducted including laboring women with term pregnancy, maximum dilation of 8 cm, live fetus in cephalic vertex presentation. Women with bleeding disorders of pregnancy, indication for caesarean section and those without capacity to consent and without legal guardians will be excluded. Primary outcomes will be frequency of episiotomy, delivery duration, frequency of spontaneous lacerations and perineal trauma, frequency of instrumental delivery, postpartum blood loss, need for perineal suturing, number of sutures, Apgar scores at one and five minutes, need for neonatal resuscitation and pH in cord blood. As secondary outcomes frequency complications of perineal suturing, postpartum perineal pain, maternal satisfaction, neonatal morbidity and admission newborn in NICU will be assessed. Women will be invited to participate and those who agree will sign the consent form and will be then assigned to a protocol of not conducting episiotomy (experimental group) or to a group that episiotomy is performed selectively according to the judgment of the provider of care delivery (control Group). The present study was approved by IMIP’s Research Ethics Committee.

**Trial Registration:**

Clinical Trials Register under the number and was registered in ClinicalTrials.gov under the number NCT02178111.

## Introduction

Despite all available evidence corroborating the selective use of episiotomy and the recommendation of NOT to perform routine episiotomies, questions remain about what are the real indications to perform episiotomy in modern obstetrical practice [1].

American College of Obstetricians and Gynecologists (ACOG) guidelines state that “the best available data do not support the liberal or routine use of episiotomy. However, there is a role for episiotomy for maternal or fetal indications such as avoiding severe maternal lacerations or to facilitate difficult births” [2]. The World Health Organization recommends an episiotomy rate of 10% as “a good goal to pursue” [3], based on the data obtained in the English randomized controlled trial published in 1984 [4].

In the Cochrane systematic review, a question arises: What would actually be the indications of episiotomy? Some situations are placed such as preterm delivery, breech delivery, macrosomia, shoulder dystocia, instrumental deliveries (forceps or vacuum extraction), non-reassuring fetal heart rate and rigid perineum or threat of severe perineal rupture [5]. However, these situations have been questioned as an indication of episiotomy and clearly this issue needs to be further studied in randomized clinical trials [1]. While it is clear that routine episiotomy should be avoided, there is no solid evidence corroborating ANY indication of episiotomy. The benefits of the use of episiotomy in these selected conditions remain controversial [6].

Recently, it has been suggested that episiotomy should never be performed. An original recommendation for performing episiotomy was famously summarized by Scott (2005) [7]: “Don’t just do something, sit there!”. In a study including 168,077 singleton vaginal deliveries in the Soroka University Medical Center, Israel (2012), mediolateral episiotomy was found to be an independent risk factor for 3rd or 4th degree perineal tears, even in critical conditions such as shoulder dystocia, instrumental deliveries, occiput-posterior position, fetal macrosomia, and non-reassuring fetal heart rate. The rates of episiotomy in this Hospital declined from more 30% in 90’s to less than 5% in 2010 [8].

With a protocol of not conducting combined with episiotomy perineal protection strategies, Amorim et al. found an intact perineum rate around 60% and only 23% of pregnant women in need of sutures that have not undergone episiotomy [9,10]. However, this was a non-controlled study with an isolated sample and the authors suggest the need for randomized clinical trials comparing a policy of not conducting episiotomy with the policy of selective episiotomy.

Reviewing the Medline, Lilacs/SciELO, EMBASE, Scopus, and the Cochrane Library databases using the keywords “episiotomy” and “vaginal delivery” in Portuguese, English and Spanish, with filters “randomized clinical trial” and “systematic review”, no clinical trial or clinical trial protocol addressing the non-performance of episiotomy care for vaginal delivery was found.

Thus, the present study will be conducted in order to compare maternal and perinatal outcomes in women undergoing a protocol of not conducting episiotomy vs. selective episiotomy.

### Objectives and hypothesis

The overall objective is to compare a protocol of not conducting episiotomy vs. selective episiotomy in a randomized clinical trial. The hypothesis is that never conducting a episiotomy is superior to using episiotomies in a selective manor.

#### ***Specific objectives***

*In pregnant women randomized to a protocol of not conducting an episiotomy vs. selective episiotomy*,*compare*:

### Primary maternal outcomes

• Duration of the second stage of labor;

• Frequency of episiotomy;

• Indication of episiotomy (imminent severe perineal rupture, instrumental delivery, shoulder dystocia, prolonged second stage of labor and non-reassuring fetal heart rate);

• Frequency of spontaneous lacerations;

• Frequency of instrumental delivery;

• Frequency of perineal trauma;

• Blood loss at delivery;

• Perineal need for suturing;

• Number of sutures;

### Primary perinatal outcomes

• Apgar scores at 1 and 5 minutes;

• Need for neonatal resuscitation;

• Cord blood pH at birth;

### Secondary maternal outcomes

• Frequency of severe perineal trauma;

• Perineal suturing complications;

• Perineal pain after childbirth;

• Maternal satisfaction;

### Secondary perinatal outcomes

• Neonatal morbidity

• Newborn admission to the NICU.

#### ***Main hypothesis***

In women randomized to a protocol of not conducting as episiotomy, in comparison to a selective episiotomy protocol:

• Duration of the second and frequency of spontaneous lacerations and perineal trauma is the same;

• Blood loss at delivery is lower, as is the need for suturing and also the number of sutures;

• Apgar scores at 1 and 5 minutes, need for neonatal resuscitation and cord blood pH at birth is the same in both groups.

• Frequency of severe perineal trauma, complications of perineal suturing, and perineal pain after childbirth is lower.

• Maternal satisfaction is higher.

• Neonatal morbidity and newborn admission to the NICU is similar.

## Methods/design

### Study design

The present study is an open label randomized controlled trial.

### Study population and location

The study population will comprehend all women in labor hospitalized in IMIP during the data collection period.

### Eligibility criteria

Laboring women with term pregnancy, maximum dilation of 8 cm, live fetus in cephalic vertex presentation will be included and women with bleeding disorders of pregnancy, immediate indication for caesarean section and women without capacity to consent and without legal guardians will be excluded.

### Procedures for selecting participants and randomization

Eligible patients will be invited to participate and those who agree will be included in the study and then will be allocated to either a protocol of not conducting an episiotomy or a protocol of selective episiotomy, according to a random list of numbers generated by the Random Allocation Software Ispharan Iran, version 1.0. This list of randomization will be provided by the statistician to researcher who will be responsible for preparing the envelopes containing instructions regarding which group the patient is allocated to (A: Intervention= > not to use episiotomy or B: Control= > selective episiotomy). This procedure will be followed in order to guarantee the concealment of allocation of patients in both arms. Patients and medical staff will not be blind to the intervention condition, but the researcher and the statistician performing the analysis will be blinded to the meaning of the letters A or B to which participants will be allocated.

During the observation period the birth attendants will conduct delivery of baby and placenta according to routine practice, except for the instruction about the use of episiotomy. Birth attendants will collect, using special plastic bags, blood loss after delivery and the amount will be registered. Blood from the cord will be collected to perform pH analysis, and suture of any lacerations that may occur will be performed according to their clinical judgment. After 24 hours of delivery, women will be interviewed and perineal pain will be evaluated using a visual pain scale and women’s satisfaction will also be assessed. During the observation period, if before birth occurs, there is an indication of cesarean delivery, the women will be excluded from the study.

### Sample size calculation

The sample size was calculated on the Open Epi 2.3 (Atlanta, GA), with an expected rate of episiotomies in the protocol group of not conducting episiotomy of 1% versus a 10% rate of episiotomies in the group of episiotomy selective [3,4]. For a power of 80% and a confidence level of 95%, 200 women would be needed, a figure that has increased by 20% to compensate for eventual losses after randomization, so that 240 women will be randomized.

### Variables

#### ***Independent variable: selective versus never episiotomy***

In the group of selective episiotomy the assistant doctor or the nurse can opt for performing an episiotomy according their clinical judgment in situations where the literature suggests that episiotomy could confer some benefit (imminent severe perineal rupture, instrumental delivery, shoulder dystocia, prolonged second stage of labor and non-reassuring fetal heart rate).

In the group of not performing episiotomy the assistant professional will assume that NEVER episiotomy is needed and avoid carrying it out, except for reasons of force majeure (clinical judgment referring to the absolute need for episiotomy).

#### ***Dependent variables***

Duration of the second stage of labor; Frequency of episiotomy; Indication of episiotomy (imminent severe perineal rupture, instrumental delivery, shoulder dystocia, prolonged second stage of labor and non-reassuring fetal heart rate); Frequency of spontaneous lacerations; Frequency of instrumental delivery; Frequency of perineal trauma; Blood loss at delivery; Perineal need for suturing; Number of sutures; Apgar scores at 1 and 5 minutes; Need for neonatal resuscitation; Cord blood pH at birth; Frequency of severe perineal trauma; complications of perineal suturing; Perineal pain after childbirth; Maternal satisfaction; Neonatal morbidity; Newborn admission to the NICU.

### Main outcomes

• Duration of the second stage of labor: time elapsed from the beginning of the second period of delivery (full dilation and maternal pushes) until the delivery of the newborn.

• Blood loss at delivery: volume of blood collected after the delivery of the child, until the first hour after delivery, in a plastic bag placed under the women.

• Apgar scores at 1 and 5 minutes: Apgar scores recorded in the first and fifth minutes after birth, as rated by the neonatologist.

• Need for neonatal resuscitation: need of any resuscitation of the newborn.

• Cord blood pH at birth: value of blood pH collected from the umbilical cord immediately after birth (considered normal or greater value 7.2).

• Severe perineal trauma: presence of perineal trauma grades III and IV. Namely, grade III perineal trauma occurs when it reaches the rectal muscle, resulting in partial or complete tearing of the anal sphincter. The grade IV laceration light exposes the rectum, reaching beyond the muscle, the mucosa retal [6]. These lacerations may be spontaneous or associated with episiotomy.

### Data collection procedures

#### ***Data collection***

For data collection, a pre-coded standard form will be used for data entry on the computer. After identifying the patients who are according to the eligibility criteria, using a specific check list, and who agree to participate in the study and sign the informed consent form, information will be collected and filled in the form. The checklist, as well as the form will be completed by the researchers.

Upon completion, the forms will be rigorously reviewed by the researchers to cross-check the information collected with the information contained in the records. Procedures for quality control, such as reviewing the completed forms and check manually typing will be adopted. A first quality control of data collection should be done before and during the typing of electronic records, to identify possible inconsistencies in the data researcher. The second quality control will check the compatibility between the physical archived records and data contained in electronic forms. The data will be entered in a specific database, created using the Epi Info statistical program 7.1.4. Each month the database will be reviewed by the principal investigator, getting listing of variables and correcting any inconsistencies or missing information from the query to the forms.

### Data analysis plan

**Figure 1:**
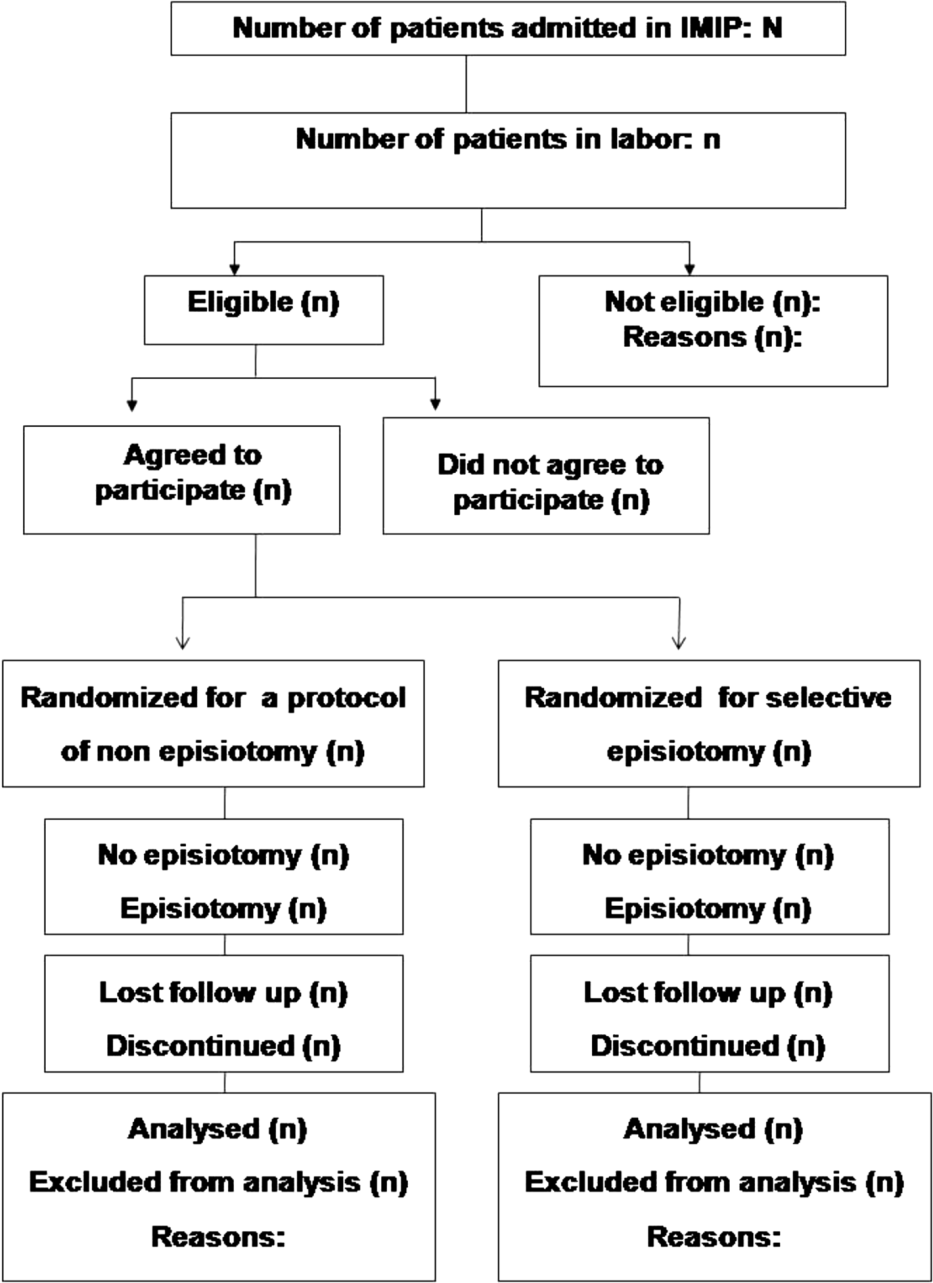
**Study design and population (CONSORT, 2010) **[6]**.**

The data analysis will be performed using the public domain software Epi Info version 7.1.4 (Centers for Disease Control and Prevention, Atlanta, GA), or the newest available version under the intention to treat principle. The statistician will remain blind to the meaning of the Groups A or B to which patients are allocated until the tables and the analysis concluded. The approach for analysis will be that showed in Figure 1 using an intention-to-treat strategy and following the correspondent recommendations from the CONSORT statement [11]. The characteristics of the participants in each group will be compared with Student’s *t* test for continuous variables with normal distribution and Mann–Whitney U test for discrete and ordinal variables or those with non-normal distribution. Categorical variables will be compared with Pearson’s χ [2] test or Fisher’s exact test, as appropriate. P values for all tests will be two tailed at a 5% level of significance. Risk ratios and their 95% confidence intervals will be calculated as a measure of relative risk. The number needed to treat (NNT) and its 95% confidence interval will be calculated for the outcomes in which a beneficial effect of not performing an episiotomy is achieved, using the EBM calculator [http://moosenose.com/EBCalculator.htm]; in case of adverse effects the number needed to harm (NNH) and its 95% confidence will also be calculated.

### Quality control

The researchers will maintain a record of problems occurred during the study and any doubt should be solved with the Steering Committee.

### Ethical issues

The original protocol of this research proposal has already obtained approval of the local Institutional Review Board from the coordinating center (IMIP, Recife, Brazil), and of the National Committee for Ethics in Research (CONEP) of the Brazilian Ministry of Health, under the number 114993. The protocol also was published in the Clinical Trials Register under the number NCT02178111(http://clinicaltrials.gov/ct2/show/NCT02178111).

Patients in labor will only be included if they agree to participate and sign the informed consent. All principles related to research in human beings established by the Brazilian National Health Council according to the Declaration of Helsinki will be followed. The confidentiality on women’s data and medical care will be ensured regardless of whether they participate in the study or not.

## Discussion

### Technical and scientific contributions of the study

Although is well-recognized that episiotomy should not be routinely performed, the actual indications for its realization remain to be established. The procedure was introduced in Obstetrics with no evidence of its claimed beneficial effects for both mothers and their neonates [6,12].

Despite consistent evidence against its indiscriminate realization (Cochrane), in some places episiotomy is still routinely performed and the most recent study published in Brazil demonstrated a rate of 54% in our country [13]. More than five times higher than the maximum rate recommended by the World Health Organization, this rate implies that a lot of health professionals (especially doctors, because in current obstetric model in Brazil the deliveries are attended mostly by doctors) continue to systematically perform this procedure.

Moreover, routine episiotomy is now considered to be a form of obstetric violence, especially when performed without an informed consent. A relatively new term, “obstetric violence” describes a situation in which any form of labor is considered pathological, when a woman is automatically transformed into a patient and routine medical and pharmacological procedures are conducted without allowing her to make decisions regarding her own body [14].

In this context, it is important to define whether episiotomy is really needed in any situation and what are their true indications. This study may help to clarify this point bringing evidence about potential benefits and possible risks of never performing an episiotomy, something that has already been claimed by the women’s movement.

In addition to this significant scientific contribution it also addresses issues of reproductive rights and may bring important contribution in terms of health care costs. According to Belizan et al. a saving between US$ 6.50 and 12.50 was estimated every vaginal birth without episiotomy in the public sector (a figure including only costs of suture materials). They estimated in their original work a saving for Brazil ranging from US$ 15 to 30 million [15].

## Abbreviations

IMIP: Instituto de Medicina Integral Prof. Fernando Figueira; WHO: World Health Organization; NICU: Neonatal intensive care unit; ACOG: American College of Obstetricians and Gynecologists; NNH: Number needed to harm; NNT: Number needed to treat; CONEP: National Committee for Ethics in Research of the Brazilian Ministry of Health; CNPq: National Counsel of Technological and Scientific Development.

## Competing interests

The authors declare that they have no competing interests.

## Authors’ contributions

The first version of this protocol was drafted by MA and LK. IM and IC revised the final complete version of the protocol. All authors have made substantive intellectual contributions to the manuscript and read and approved its final version.
